# Sticky Bone as a New Type of Autologous Bone Grafting in Schatzker Type II Tibial Plateau Fracture Case Report

**DOI:** 10.3390/life14081042

**Published:** 2024-08-21

**Authors:** Stefan Iulian Stanciugelu, Jenel Marian Patrascu, Sorin Florescu, Catalin Marian

**Affiliations:** 1 Doctoral School, “Victor Babes” University of Medicine and Pharmacy Timisoara, Eftimie Murgu Square 2, 300041 Timisoara, Romania; stefan.stanciugelu@umft.ro; 2 “Pius Brinzeu” Emergency Clinical County Hospital, Bld. Liviu Rebreanu, No. 156, 300723 Timisoara, Romania; florescu.sorin@umft.ro; 3 Orthopedics II Research Center, “Pius Brinzeu” Emergency Clinical County Hospital, Bld. Liviu Rebreanu, No. 156, 300723 Timisoara, Romania; 4 Prof. Univ. Dr. Teodor Șora Research Centre, Timisoara, Eftimie Murgu Square 2, 300041 Timisoara, Romania; 5 Department of Orthopedics and Traumatology, “Victor Babes” University of Medicine and Pharmacy Timisoara, 300041 Timisoara, Romania; 6 Department of Biochemistry and Pharmacology, “Victor Babes” University of Medicine and Pharmacy, Eftimie Murgu Square 2, 300041 Timisoara, Romania; cmarian@umft.ro; 7 Center for Complex Networks Science, “Victor Babes” University of Medicine and Pharmacy, Eftimie Murgu Square 2, 300041 Timisoara, Romania

**Keywords:** tibial plateau fracture, sticky bone, autograft, platelet-rich plasma, platelet-rich fibrin, knee, new techniques

## Abstract

Background: Schatzker type II fractures usually need to be grafted. Autograft bone from the iliac crest represents the gold standard, but it comes with high rates of morbidity on the donor side. Sticky bone is one of the regenerative therapies that aims to find new solutions to treat bone defects and to overcome the limitation of conventional options regarding bone grafts, due to their content in growth factors, which offer osteo-induction and osteo-conduction properties. Notably, regenerative dentistry has been at the forefront of applying these products in bone regeneration, demonstrating that PRF produces a highly promising “sticky bone” when combined with bone chips. To the best of our knowledge, this grafting technique has not been used in the orthopedic field to date. Methods: The subject was a 53-year-old woman with a Schatzker type II tibial plateau fracture, for which a new autologous bone grafting technique, i.e., sticky bone, was used for the treatment of the fracture. Results: This case reports the effectiveness of sticky bone as autologous bone graft used in Shatzker type II tibial plateau fracture. As an indispensable component of regenerative medicine, it seems to be an ideal biologic graft with a fibrin-rich structure that provides effective treatment in impressed tibial plateau fractures. Conclusion: Sticky bone showed promising results and should be considered in the future as an appropriate bone implant.

## 1. Introduction

Tibial plateau fractures account for approximately 1–2% of all fractures, with an incidence of about 10.3 per 100,000 people annually. These injuries typically involve the proximal tibia and are often associated with soft tissue damage. The average patient age is around 52 years, showing a bimodal distribution: younger males in their 40s often suffer from high-energy trauma, while older females in their 70s usually sustain these fractures from low-energy falls [1]. Impression fractures of the external tibial plateau are common in these patients, where the metaphyseal bone is often osteoporotic. The primary therapeutic objectives in the surgical management of tibial plateau fractures include the precise restoration of joint anatomy through the accurate alignment of joint surfaces, thereby ensuring both functional integrity and stability of the joint. This involves the anatomical reduction of displaced bone fragments to their original positions. Achieving correct alignment of the joint surfaces is crucial for minimizing the risk of post-traumatic osteoarthritis and for maintaining optimal long-term knee joint functionality [2]. Additionally, the prevention of complications, such as postoperative infections and thrombosis, the establishment of an optimal healing environment using appropriate internal fixation techniques (e.g., screws, plates, external fixators), and the implementation of a comprehensive postoperative rehabilitation protocol are essential components of the therapeutic strategy. It is important to note, however, that, while anatomical reduction is a critical goal, its relative importance may be secondary to functional outcomes in certain clinical scenarios [3,4,5]. To achieve these objectives, procedures involving bone grafts are often necessary.

Many options for graft filler are used to address bone defects, with autologous iliac bone graft being one of the most common and considered a gold standard procedure. However, complications, such as pain, hematoma, infection, or nerve injury, may occur in up to 39% of cases [6]. Synthetic bone graft substitutes are addressing these complications and have gained significant popularity in recent years due to their efficacy in facilitating the healing of metaphyseal bone, which typically heals well [6,7]. However, synthetic substitutes are not cost-effective, and the limited availability of autogenous bone grafts underscores the pressing need to identify an “ideal” graft. This necessity drives the exploration of new directions in substitute bone grafts, which have the potential to become leading solutions in the future.

Recent studies on autologous platelet-rich blood products for tissue and bone regeneration have gained significant attention in regenerative medicine, especially in dentistry [8,9]. These products address issues like ridge deficiencies and inadequate bone for dental implants. Two main types of platelet concentrates are used: first-generation platelet-rich plasma (PRP) with anticoagulants, and second-generation platelet-rich fibrin (PRF) without anticoagulants [10,11]. Platelet and leukocyte-rich fibrin (L-PRF) membranes are crucial for regeneration due to their high platelet concentration, which releases growth factors, such as PDGF, EGF, VEGF, and TGF. Optimal therapeutic effects are achieved with platelet counts 4–5 times higher than normal, obtained via centrifugation [12].

To create a pliable, flexible, and biologically active bio-scaffold, various types of bone grafts have incorporated PRF membranes, resulting in the so-called “sticky bone” or “gummy bone”. In 2010, Sohn et al. introduced the “Sticky Bone” concept as a solidified bone graft embedded in a fibrin network. This bone graft matrix is rich in growth factors and is constructed using autologous fibrin glue (AFG). The primary characteristic of sticky bone is its adherence to surrounding tissues, which eliminates the risk of graft loss and accelerates healing through its rich content of growth factors [13,14,15].

Herein, we describe a case of Schatzker type II tibial plateau fracture, treated with autologous sticky bone (mix of PRF, PRP and cortical bone) and, to the best of our knowledge, no similar clinical procedure of autologous bone grafting has been described to be used in the orthopedic field so far. The objective of this report is to introduce this technique, which is well established in other fields, as a new type of autologous bone grafting in the orthopedic field, used as a bio-scaffold for the treatment of bone defects, and to describe the surgical technique involved.

## 2. Clinical Case Presentation

A 53-year-old female presented to the emergency room (ER) with complaints of pain and limited movement in her right knee following a fall in her yard. Upon physical examination, swelling of the right knee and positive local tenderness were detected. Plain radiographs and computed tomography (CT) revealed a collapse-type fracture of the lateral tibial plateau, classified as Schatzker II. She underwent conservative treatment for five days before being transferred to our department. Surgery was performed on the second day after her admission to the orthopedic department.

The patient provided written informed consent, and the case report was approved by the Ethics Committee for Scientific Research of the Timisoara County Emergency Hospital (Approval No. 463/19.04.2024).

**Figure 1:**
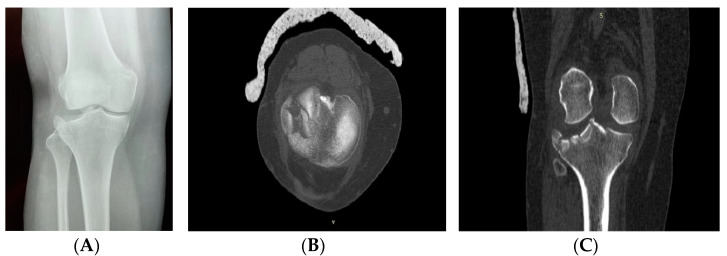
Pre-operative imagistic investigations of the right knee. (**A**) Anteroposterior plain radiographs of the right knee showing lateral tibial plateau fracture. (**B**) Initial CT scan coronal plan of the right knee showing the comminution of the lateral tibial plateau. (**C**) Initial CT scan axal slice of the right knee showing the impression of the fracture in the lateral tibial plateau.

Imagistic investigations of the right knee can be seen in Figure 1A–C.

Pre-operatively, the soft tissue surrounding the knee was thoroughly evaluated, and the patient underwent antiseptic showers with Betadine soap. Intra-operatively, an anterolateral approach was employed, involving a straight incision approximately 5 cm in length at the level of the patella, lateral to the patellar tendon, extending distally to the articular line and anteriorly to the level of Gerdy’s tubercle. The deep fascia was incised anterior to the iliotibial tract, starting from Gerdy’s tubercle and extending distally. The proximal attachment of the tibialis anterior muscle was released to expose the bone. A sub-meniscal arthrotomy was performed to access the joint. Suture threads (2.0 mm) were placed through the meniscus for subsequent reattachment. The fracture site was exposed, revealing compression of the articular cartilage.

### 2.1. Advanced Platelets Rich Fibrin (A-PRF) Membrane and Sticky Bone Preparation

#### Blood Collection

Proper hydration of the patient was maintained throughout the day of surgery. Venous blood was collected via venipuncture of the cubital vein, with 60 mL of venous blood drawn and placed into six 10 mL vacutainer tubes: four tubes for PRF, one for PRP, and one for fibrin glue, using the Pro Cell kit (PRF Process Choukroun, Nice, France), following the manufacturer’s instructions. To obtain Platelet-Rich Fibrin (PRF) membranes, venous blood was collected in four A-PRF (Advanced Platelet-Rich Fibrin) Choukroun 10 mL tubes, and then was centrifuged at 800 rpm for 12 min, in an XC 2000 medical centrifuge. After centrifugation, the PRF membranes were extracted under sterile conditions and placed in a specific press for 8–10 min. Cell separation for PRP was achieved by centrifuging the blood in a specific 10 mL vacutainer tube with separation gel for 10 min using the same centrifuge. Fibrin glue was obtained by centrifugation at 2700 rpm for 3 min. The components of the sticky bones and the centrifugation process can be seen in Table 1.

To obtain the sticky bone augmentation block, bone chips were harvested from the proximal one-third of the tibia using the OT7S micro-saw tip. The final mixture contained bone chips in a ratio of 60/40, comprising 60% bone chips and 40% a combination of four A-PRF membranes, a few drops of PRP, and fibrin glue.

**Figure 2:**
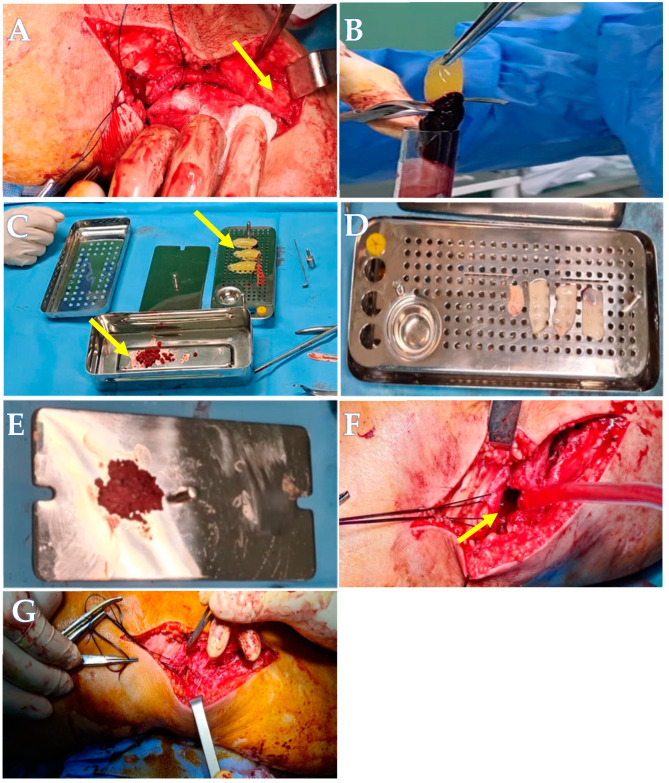
The sticky bone procedure. (**A**) Harvest area of the bone chips. (**B**) Extraction of the PRF membrane. (**C**) PRF membrane and bone chips. (**D**) PRF membrane after compressing with metal cover. (**E**) Final product, sticky bone. (**F**) Tibial defect. (**G**) Tibial defect filled with sticky bone.

Reduction of the articular surface was achieved using a curved impactor. The resulting bone defect was filled with the sticky bone graft and stabilized with two screws. The wound was then closed, as seen in Figure 2A–G.

**Figure 3:**
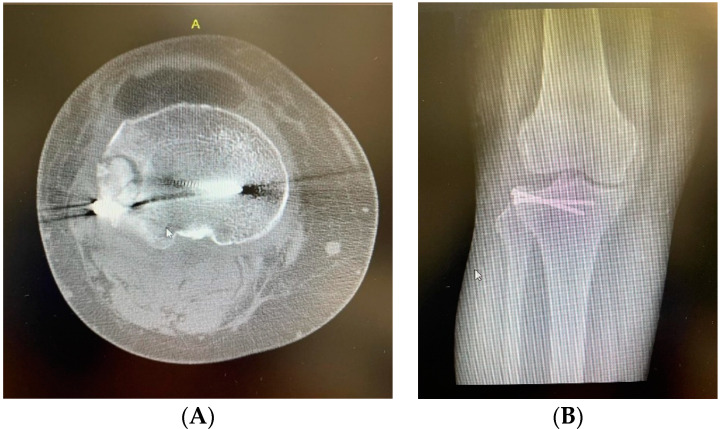
Post-operative CT scan of the right knee. (**A**) Post-operative AP CT scan of the right knee showing the reduction and fixation of the fracture after augmentation with sticky bone. (**B**) Post-operative AP plain radiograph of the right knee showing the reduction and fixation of the fracture after augmentation with sticky bone.

The patient was discharged 48 h postoperatively. Functional treatment commenced on the second postoperative day, adhering to a rehabilitation program that included continuous passive motion (CPM), static quadriceps exercises, and active movements of the knee and ankle. Weight bearing was restricted for 10 weeks postoperatively. At a follow-up appointment, 13 months after surgery, the patient exhibited a full range of motion in the knee joint without pain. The postoperative scar demonstrated a normal appearance, with no signs of inflammation, as observed in Figure 3A,B.

**Figure 4:**
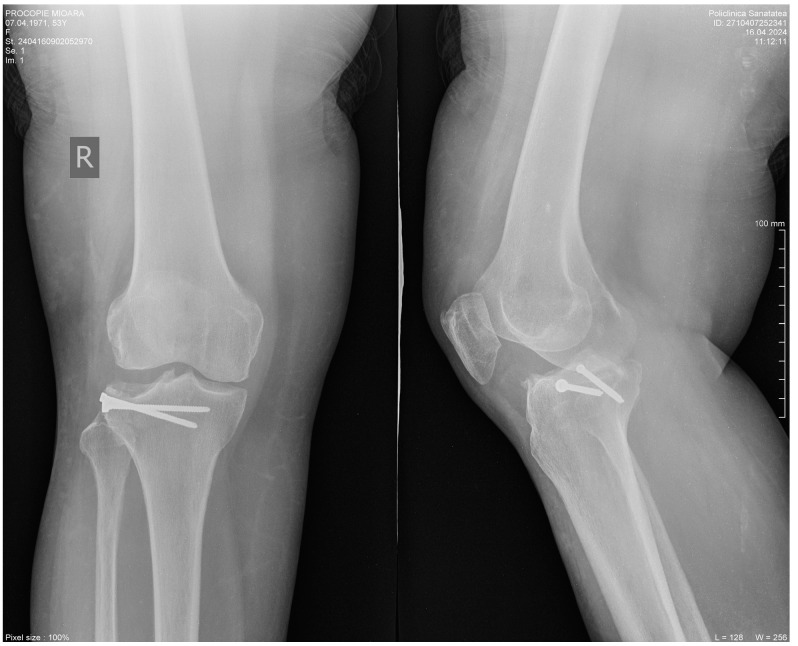
Radiological follow-up, at 13 months post-operatively.

Radiological follow-up, at 13 months post-operatively, presents without signs of arthrosis or subchondral suffering of the bone at the level of the external tibial plateau. The initial stabilization was maintained (Figure 4).

**Figure 5:**
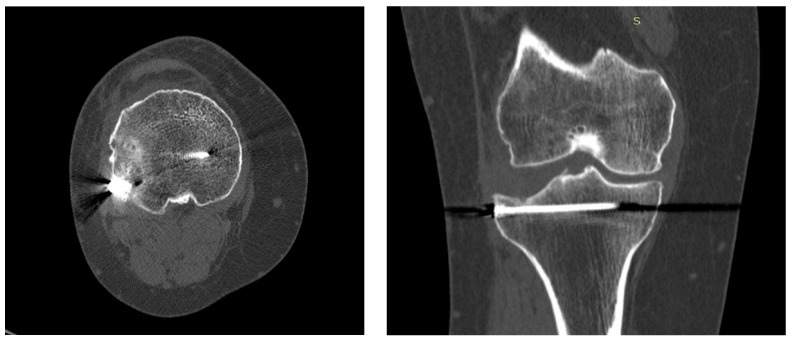
Axial (**left**) and coronal (**right**) computed tomography of the right knee, at 18 months follow-up.

The axial CT image (Figure 5, left) shows a proximal tibial plateau fracture stabilized with a metallic screw. Bone margins are well-defined with no signs of lysis or severe demineralization. The joint space is slightly reduced, and peri-screw sclerosis indicates ongoing healing. Overall, the image suggests successful surgical stabilization with evidence of bone consolidation.

The coronal CT image (Figure 5, right) shows a tibial plateau fracture stabilized with a metallic screw. Bone margins are well-defined, with no signs of resorption or complications. Peri-screw sclerosis indicates ongoing healing, and the joint space is preserved. Overall, the image suggests favorable fracture healing with stable fixation.

The axial CT image shows a proximal tibial plateau fracture stabilized with a metallic screw. Bone margins are well-defined with no signs of lysis or severe demineralization. The joint space is slightly reduced, and peri-screw sclerosis indicates ongoing healing. Overall, the image suggests successful surgical stabilization with evidence of bone consolidation.

Our patient presented with a type II Shatzker tibial plateau fracture, which was comminuted, as evidenced by CT imaging, and occurring in bone of good quality. Complex or displaced fractures necessitate robust and stable fixation, often achieved through the use of screw plates, which provide essential stability throughout the healing process and prevent displacement of the bone fragments under load. This is particularly critical in fractures involving large joint surfaces [16].

Considering the patient’s normal body weight, good bone quality, and the supportive nature of the graft used, which adequately maintains the position of the displaced bone fragments during the non-weight-bearing period, we concluded that additional fixation with two screws would be sufficient to secure the fracture site during the healing process. Marco Verona reports more satisfactory outcomes with minimally invasive, arthroscopically assisted treatment using internal fixation with cannulated screws, compared to open reduction and internal fixation for Schatzker type I–III fractures.

## 3. Discussion and Review of the Literature

The present case report aims to introduce sticky bone in the orthopedic field as a novel biological autologous bone graft characterized by a fibrin-rich structure and significant regenerative properties due to its content of growth factors. Schatzker type II fractures are depression fractures of the external tibial plateau, with treatment primarily focused on restoring the joint surface. Despite being associated with high morbidity rates, bone graft harvested from the iliac crest is considered the gold standard for open procedures due to its superior mechanical and biological properties. Alternatively, bone allografts and bone substitutes can be used, but they are not always available and are often not cost-effective [17].

Our approach to addressing the created bone defect involved using a biological graft composed of PRF membranes, PRP, fibrin glue, and bone chips, which possess osteogenic and plastic properties. The PRF technique has demonstrated numerous advantages over other treatment methods: it does not require advanced technical skills, biochemical modifications are minimal, it is cost-effective, fibrin meshes incorporate more circulating cytokines, polymerization time is slower, healing is accelerated, and structural integrity is improved [18,19].

Due to its potential as a method for tissue regeneration, PRP and plasma rich in growth factors, as first-generation concentrates, have been recommended by many authors for use in various clinical cases. In contrast, leukocyte-platelet-rich fibrin (L-PRF) and advanced platelet-rich fibrin (A-PRF) have demonstrated superior biological properties in musculoskeletal regeneration. The physiological fibrin matrix included in these more recent platelet concentrates functions as a scaffold with a three-dimensional (3D) architecture. Additionally, it promotes angiogenic, chondrogenic, and osteogenic processes by facilitating intercellular signaling and movement [20].

The CT images of the knee at 18 months follow-up demonstrate a surgically stabilized tibial plateau fracture with metallic screws in place. Bone margins are well-defined without signs of lysis or significant demineralization, and peri-screw sclerosis suggests ongoing bone healing. Preclinical and clinical studies indicate that systemic bone loss initiates shortly after a fracture and can persist for several years in humans. Notably, both bone quantity and quality may not fully recover to pre-fracture levels, particularly in older individuals. This highlights the need to better understand the processes behind this bone loss to help reduce the risk of future fractures [21]. Overall, the radiologic findings indicate successful fracture stabilization and evidence of bone consolidation, though further clinical and imaging follow-up is recommended to ensure continued favorable healing.

Simonpieri and his colleagues have introduced the concept of “natural bone regeneration” and attempted to achieve reconstruction of alveolar ridges at both gingival and bone levels. For surgeries requiring a higher quantity of L-PRF, multiple centrifuge tubes can be utilized. They have also observed that using multiple PRF layers may yield favorable results in immediate implant techniques [22]. A higher number of proteins can be accumulated by using the new PRF, which contains more growth factors [23]. Gassling et al. concluded that the PRF technique provides better support for cell proliferation than collagen. In vitro studies have used PRF membranes to cultivate periosteal cells, a procedure essential for gaining experience in bone engineering [24,25]. The PRF technique has shown good results in treating periodontal infra-bone defects and has been successfully used in the augmentation of the maxillary sinus floor and in closing sinus perforations. It has been found that combining PRF with bone grafts can reduce the quantity of bone substitute needed and accelerate revascularization due to PRF’s angiogenic properties [26].

Everts et al. revealed that bone particles and osteoblasts form a very strong structure when combined with thrombin and platelets, with PRF fragments serving as a biological connector between bone particles [12]. The new fibrin glue, which acts as an efficient adhesive, can perfectly imitate the normal coagulation process. This material consists of fibrinogen and thrombin, both crucial for the coagulation process. Fibrinogen can be either heterologous or autologous, the latter being derived from cryoprecipitate or plasma. In the process of fibrin formation, prothrombin is the initial factor, which, upon cleavage, forms thrombin. Thrombin is a protease that converts fibrinogen into the complex fibrin, which forms the blood clot during a vascular injury [27].

Fibrin glue can be procured in two versions: commercial and autologous. The commercial version contains homologous and xenogeneic components, while the autologous version is obtained from the patient’s venous blood. The autologous alternative is safer because it significantly reduces the risks of contamination and immunological responses. One disadvantage of the autologous version is its low reproducibility, which depends on the individual patient. Fibrin glue can be used in various ways, as hemostatic agent, sealant, or adhesive. Many authors have utilized tissue adhesives in surgery for purposes including bone regeneration, closing osteochondral defects by attaching a graft of periosteal tissue, and repairing bone and cartilage. [27].

These adhesives are also indicated in microsurgical techniques due to their numerous advantages: elasticity, tensile strength, and high adhesiveness to tissues [28,29]. This latter property is strongly related to the complex process of polymerization, in which thrombin and fibrinogen play crucial roles. Many studies have documented the adhesive strength of commercial fibrin sealants. In the process of obtaining fibrin glue, a special centrifuge is not required; only equipment to extract derivatives from autologous blood is necessary. [30,31]. 

At the site of an injury, fibrin creates a mesh to facilitate hemostasis and allows the formation of extracellular matrices. Despite its regenerative capacity, which is difficult to fully understand, bone can repair itself in many cases of defects. However, when the defects are larger, bone grafts are required [32,33]. It has been shown that autogenous and autologous materials are the best options for use in the regenerative process. Fibrin glue of autologous origin is more recommended than the commercial version due to its safety and reduced risk of immunological responses. 

Second-generation growth factors, such as PRF and other highly concentrated factors, are used specifically to activate platelets and stimulate the cross-linking of fibrin [34]. PRF has different purposes in surgery, depending on the way it is placed. The most important property is to contain the main chemical additives such as thrombin and anti-coagulant. PRF can be also used in the form of a membrane which is resorbable. This membrane is placed in different surgical sites and it triggers the mineralization of the clot beneath. Being mixed with a bone graft, the PRF membranes can also provide protection of the site and stimulate the healing of the wound and the soft tissue. Practically, they offer a support for the stem cells and the osteoprogenitor cells start to move towards to center of the bone graft. This is the primary step in the process of neo-angiogenesis [35]. In addition to serving as a biological connector, PRF also acts as a biological adhesive, maintaining the integrity of the tiny bone particles. This property facilitates easier manipulation of the graft.

The concept of sticky bone involves combining fibrin glue with a bone graft. The most important characteristics of autologous bone are its osteo-inductivity, osteo-conductivity, and osteo-genesis. [36,37]. Numerous benefits of sticky bone have been described in recent studies. The strongly interconnected fiber network of its components offers considerable plasticity, allowing the graft to be adapted into different forms to fill bone defects. Additionally, the augmentation technique with sticky bone serves to replace the metaphyseal gap and provide mechanical support until healing is achieved, typically within 6–8 weeks. [14,38]. Allografts are an important alternative to consider, especially in the treatment of large bone defects. Unfortunately, they are not always available and can increase the cost of treatment. Additionally, compared to sticky bone, allografts carry risks, such as rejection, disease transmission, inconsistent incorporation, and late resorption [39].

In a recent publication from 2022, Ghoderao et al. concluded that patients with periodontal defects treated with sticky bone showed better clinical and radiological progress at a 12-month follow-up compared to those treated with concentrated growth factor (CGF). His comparative study included 20 subjects with a total of 40 intra-bony defects [38].

Operating on a secondary site can have numerous disadvantages. Besides the high risk of infection, the cost, and the extended treatment time required, there are several other impediments. The quantity of graft available is often insufficient and easily displaceable, and the donor site is susceptible to deformation. Additionally, low vascularization can lead to poor viability, which can adversely affect the graft’s prognosis [40,41]. Taking into consideration all the possible disadvantages, the newest technology aims to manage and avoid the use of autologous grafts [42,43,44]. Organs and tissues can be created in vitro conditions by using a three-dimensional mesh, a scaffold that provides a favorable support for cells that will develop the organ. The basic conditions to be fulfilled by the scaffold would be biocompatibility, biodegradability, and the mesh should also allow a high reaction of adhesion between the cells [45,46]. All these properties must be accompanied by excellent adaptability to defect sites and flexibility, and they should not provoke any adverse reactions. Bone ingrowth is crucial for the success of the graft. Most of these properties are met by materials such as polyglycolic acid, polylactic acid, and polylactic-co-glycolic acid, which are widely used in tissue engineering. However, the residual acids released by these synthetic materials frequently result in inflammatory reactions [40].

Due to its biocompatibility and biodegradability, fibrin has proven its effectiveness as a natural scaffold, surpassing synthetic materials. It also provides high graft stability and stimulates the release of growth factors. This leads to effective cell culture on the scaffold, promoting the development of bone and cartilage [47,48,49,50].

Sticky bone consists of a bone graft combined with growth factors, with the fibrin glue being of autologous origin. Sticky bone provides good graft stability in the osseous defect and accelerates the healing process. Due to these properties, bone loss during healing is minimized [27].

In our case report, we sought to present the effectiveness of sticky bone in treating orthopedic fractures. However, several limitations of the study must be acknowledged. A primary limitation is that only a single case was treated with this type of graft. Consequently, future studies with larger sample sizes are necessary to draw definitive conclusions. The size of bone defects treated with this type of graft is contingent on the amount of bone chips that can be harvested. In future research, it will be imperative to standardize the size of bone defects that can be treated with this type of graft. Furthermore, potential assessment biases may have occurred due to follow-up evaluations being conducted by the operating surgeon.

## 4. Conclusions

This case report introduces the innovative use of “sticky bone”, a combination of autologous platelet-rich fibrin (PRF), platelet-rich plasma (PRP), and bone chips, as a bio-scaffold for treating tibial plateau fractures. The successful application in a Schatzker type II fracture demonstrates the potential of this grafting technique to enhance bone healing, provide stable fixation, and minimize bone loss during recovery. While the initial results are promising, this study’s limitations, including the single-case focus, highlight the need for further research with larger sample sizes and standardized protocols. The findings suggest that sticky bone could be a viable alternative to traditional grafting methods in orthopedic surgery, particularly when considering its biocompatibility, ease of use, and regenerative properties. Future studies are essential to validate these outcomes and explore the broader applicability of this technique in orthopedic practice.

## Figures and Tables

**Table 1:** Sticky bone components and centrifugation process.

Type of Tubes	Product	Time of Centrifugation in Min	Revolutions per Minute (rpm)
A-PRF Choukroun 10 mL	A-PRF	12	800
Specific PRP Gel separation tube	PRP	10	3500
Fibrin	Fibrin Glue	3	2700

## Data Availability

Data are contained within the article.
